# Control programming without language: automation of vitamin B_6_ analysis

**DOI:** 10.1155/S1463924681000527

**Published:** 1981

**Authors:** J. F. Brown, J. T. Vanderslice, C. Maire, S. G. Brownlee, K. K. Stewart

**Affiliations:** 1 Nutrient Composition Laboratory Beltsville Human Nutrition Research Center, Human Nutrition Science and Education Administration US Department of Agriculture Beltsville Maryland 20705 USA; 2 Nexus Associates 6500 Hanover Heights Trail Clifton VA 22024 USA


### Control programming without language: automation of vitamin $B_6$ analysis*

**J.F. Brown**, J.T. Vanderslice, C. Maire, S.G. Brownlee and K.K. Stewart**

*Nutrient Composition Laboratory, Beltsville Human Nutrition Research Center, Science and Education Administration, US Department of Agriculture, Beltsville, Maryland 20705, USA*

### Introduction

An improved and semi-automated method has recently been reported by Vanderslice and Maire for determining very low concentrations of the $B_6$ vitamers by HPLC [1]. The method is sensitive enough for the analysis of whole blood and has a detection limit of 0.01 ng/ml for the metabolite pyridoxic acid and 0.10 ng/ml for the $B_6$ vitamers. Separation of the vitamers is significantly improved over previous methods, and no interference is observed from the other water-soluble vitamins [2-5].

A Perkin-Elmer 650-40 fluorescence spectrophotometer and a Shimadzu CR-1A integrator are primary components of the analytical system. These instruments have so called "smart" features which are implemented by single keystroke commands and which provide capabilities that would be difficult or impossible to duplicate manually. The Perkin-Elmer spectrophotometer functions used for vitamin $B_6$ analysis execute changes in zero-suppression and both excitation and emission wave-lengths. The integrator was programmed to integrate and plot the analog detector signal from the spectrophotometer, print results and control elements of the HPLC system. These "intelligent" capabilities generally reduce the time and manipulative skill required of the operator.

Figure 1 illustrates the commands entered at the instrument keyboards as they were required during the manual analysis of vitamin $B_6$. The integrator requires a START command to begin integrating data and controlling the HPLC system for each sample. The operator must also change the spectrophotometer excitation and emission wave-lengths and zero-suppression four times during the 75 minute analysis to optimise detection of the individual $B_6$ vitamers. These requirements interrupt the operator's other activities and obviously prohibit unsupervised overnight operation. The need for frequent human attention during the analysis is an obstacle to full automation of the analytical system.

Automating the spectrophotometer/integrator system to analyze multiple samples without human attention was accomplished using a simple, inexpensive and non-traditional technique which can be implemented on many analytical systems without special skills. The method involves interfacing a microcomputer to the instrument keyboards and associated external servo-controls and using software that allows the microcomputer to "learn" and then imitate operator actions during an analysis. The microcomputer "learns" how to carry out the analysis simply by observing what the operator does; explicit programming is unnecessary.

** A preliminary report of this work was presented by J.F. Brown, J.T. Vanderslice and C. Maire at the 32nd Pittsburgh Conference on Analytical Chemistry and Applied Spectroscopy, March 9–13, 1981, paper number 93.*

*** Present Address: Nexus Associates, 6500 Hanover Heights Trail, Clifton, VA 22024, USA*

This paper concentrates on the microcomputer and electrical aspects of automated vitamin $B_6$ analysis; a future publication will present details of the chemistry, methods and HPLC hardware as a model system[6].

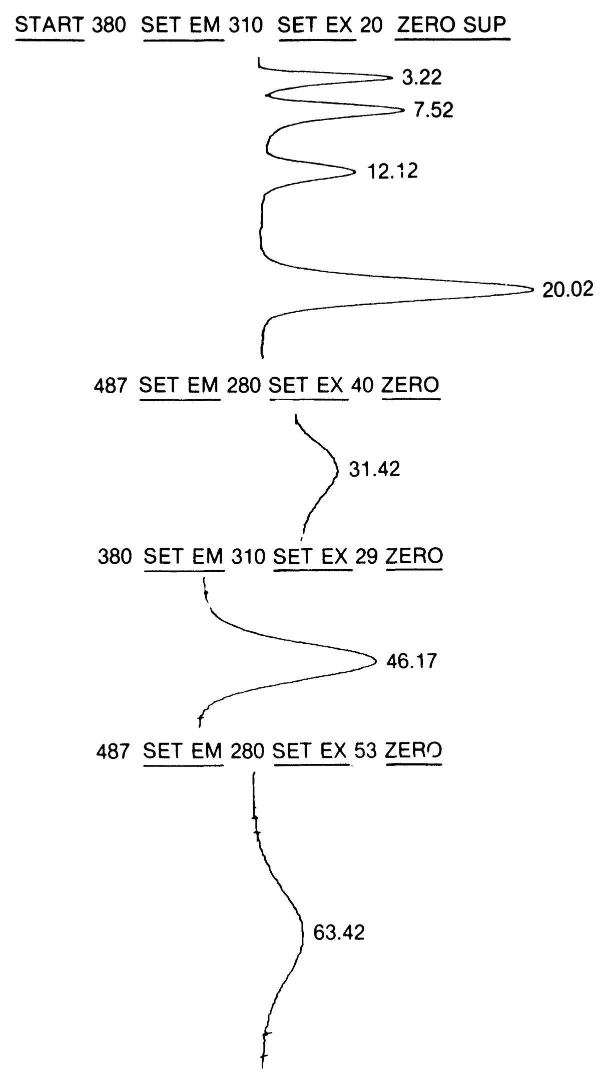

*Figure 1. Chromatogram showing commands entered on the integrator and spectrophotometer keyboards as they are required during vitamin $B_6$ analysis. START runs the integration program: SET EX, SET EM and ZERO SUP change the excitation and emission wavelengths and zero offset respectively on the spectrophotometer.*

### Hardware

Figure 2 is a diagram of the fully automated system for analysing vitamin $B_6$ using a Rockwell AIM 65 microcomputer. The AIM 65 provides synchronised control of the spectrophotometer, the integrator, and the sample and column-control valves. Standard features of the AIM 65 include a terminal-style keyboard, 20-column printer and display, serial interfaces and 16 input/output lines. 4 K bytes of random access memory and Rockwell's 8 K ROM BASIC were options selected for this application.

The method of interfacing the AIM 65 to instrument keyboards is simple and direct. Figure 3a is a schematic which represents both of the commercial instruments keyboard circuits (many instrument keyboards, in fact, use this standard design). The pull-up resistor allows a few milliamps to pass to the input of a logic gate and to a switch or key; the other side of the switch is connected to ground. The voltage at the gate input is high when the switch is open and low when the switch is closed. By enabling the logic gate, the instrument microprocessor can evaluate the state of the switch. Pressing an instrument key, therefore, simply drains a small current to ground.

The AIM 65 input/output lines are buffered and bidirectional. When they are programmed as inputs, the microprocessor can sense whether the incoming signal is high or low. The I/O buffer can also be programmed to output several milliamps at 5 V in the high state or to output a low state which will drain about 30 mA to ground. Since pressing a key on the spectrophotometer or integrator drains a few milliamps from one side of the key to ground, connecting a microcomputer I/O line to the key gives the microcomputer remote control of the instrument function which would normally be effected by manually pressing the key. In Figure 3a, that corresponds to connecting a microcomputer I/O line to point A on the instrument keyboard, which is how the AIM 65 is interfaced to the spectrophotometer and integrator. Each significant key used during the analysis was connected directly to a microcomputer I/O line. These included the integrator START key and the numbers (0–9), ZERO SUP, SET EX and SET EM keys on the spectrophotometer.

The circuit shown in Figure 3b was used to control the sample and column-control valves. Solid-state relays (SSRs) are convenient interfaces between digital equipment and high-power devices and they also minimise electrical noise to instrument detectors. The optical switch and high-power load can be activated by closing the manual switch which allows a small current to flow through the LED. Of course, the microcomputer can also control solid-state relays in the same manner in which keyboards were controlled by attaching an I/O line at point B (Figure 3b).

A parts list and detailed schematics are available upon request from the author.

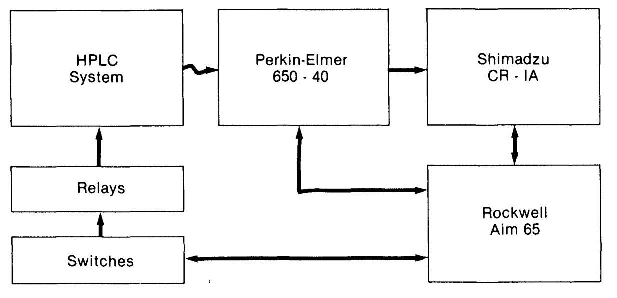

*Figure 2. Diagram of the automated chromatographic system for multi-sample analysis of vitamin $B_6$.*

### Software

Connecting the keyboard logic gates and SSRs to the AIM 65 I/O lines not only provides control of the analytical system but also allows the microcomputer, in the input mode to know the state of the analytical system. These features are the basis for controlling analytical systems without language or programming. Instead of writing microcomputer programs to control specific instruments and devices and requiring programming skills of analytical chemists, an uncomplicated program has been developed which allows the microcomputer to teach itself. The analyst need only carry out the manual procedure once with the microcomputer in the "LEARN" mode; thereafter, the procedure can be automatically repeated for any number of samples by the microcomputer. In the "LEARN" mode, the microcomputer observes the keys and switches as the analyst manually operates the instrument and

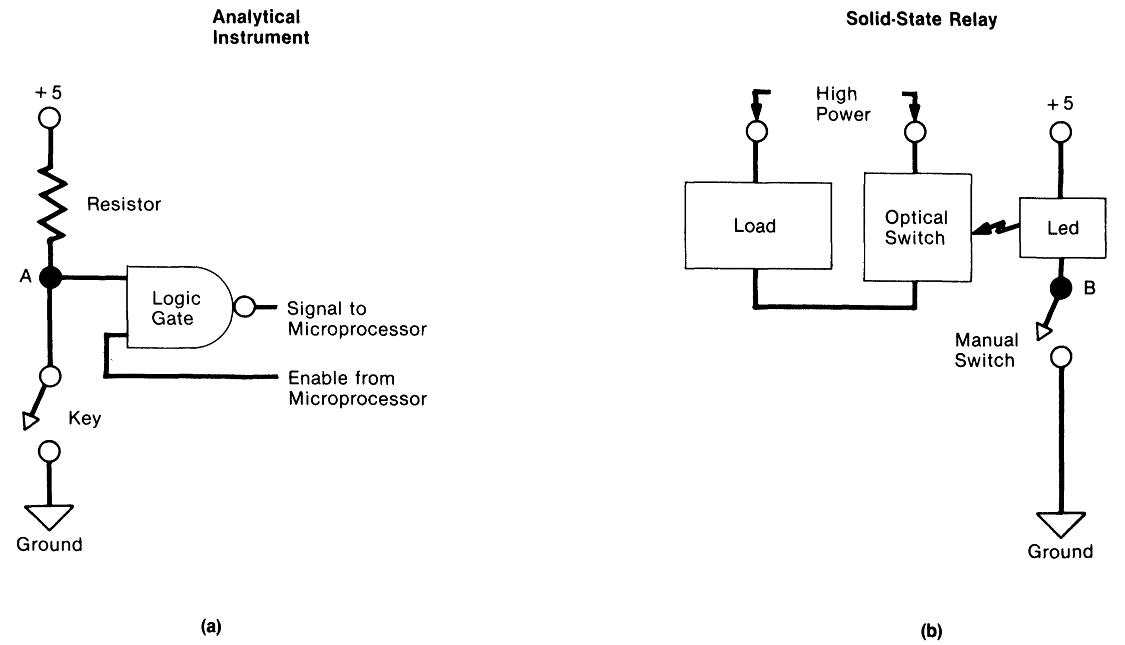

*Figure 3. Part (a) is a schematic of the integrator and spectrophotometer keyboard interface; (b) illustrates the use of solid-state relays to control high-power devices.*

makes a time-based record of user keyboard entries and device states which is stored in a method file. These instrument states can then be reproduced with appropriate time delays in the "RUN" mode. Adapting the software to different instruments and devices is unnecessary, since the key and switch correspondence to the microcomputer memory is established implicitly in the "LEARN" operation. The microcomputer has essentially only three commands: LEARN, ESCAPE, and RUN.

Figure 4a is a flow chart of the "LEARN" operation. The program index, I, and Time (I) are initialed to zero, and the instrument state is stored in the method file as State (I). Every 1/10th second, the program compares State (I) with the current instrument state to determine if a change in state has occurred. If the state is unchanged, TIME (I) is incremented (maximum time is BASIC's integer limit) and a check of the microcomputer keyboard is made for a command to stop learning (ESCAPE). If the operator has not entered ESCAPE, then another comparison is made of the current instrument state and State (I). When the operator presses an instrument key or switches on a device, and the state of the system changes, index I is incremented, Time(I) is set to zero, and the instrument state is recorded. At the end of the analysis, the ESCAPE command terminates the "LEARN" mode and causes the complete method file of I/O states and delays to be printed out.

Once a satisfactory manual analysis has been learned by the AIM 65, samples may be automatically analysed in the "RUN" mode. As shown in Figure 4b, the index is first set to zero, State (I) is output to the servos and instrument keyboards, and the microcomputer counts down from Time(I). When the time delay is finished, the program determines if the last instruction has been executed. If other instructions remain, the index is incremented and the operation continues. After the last instruction, the program repeats the instruction sequence until the last sample has been analysed.

Teaching the AIM 65 to control the spectrophotometer and integrator during a vitamin $B_6$ analysis simply involves typing "LEARN" on the AIM 65 keyboard and then manually carrying out the analysis. When the analysis is complete, pressing the ESCAPE key on the AIM 65 terminates the "LEARN" operation and prints out a record of the pro-

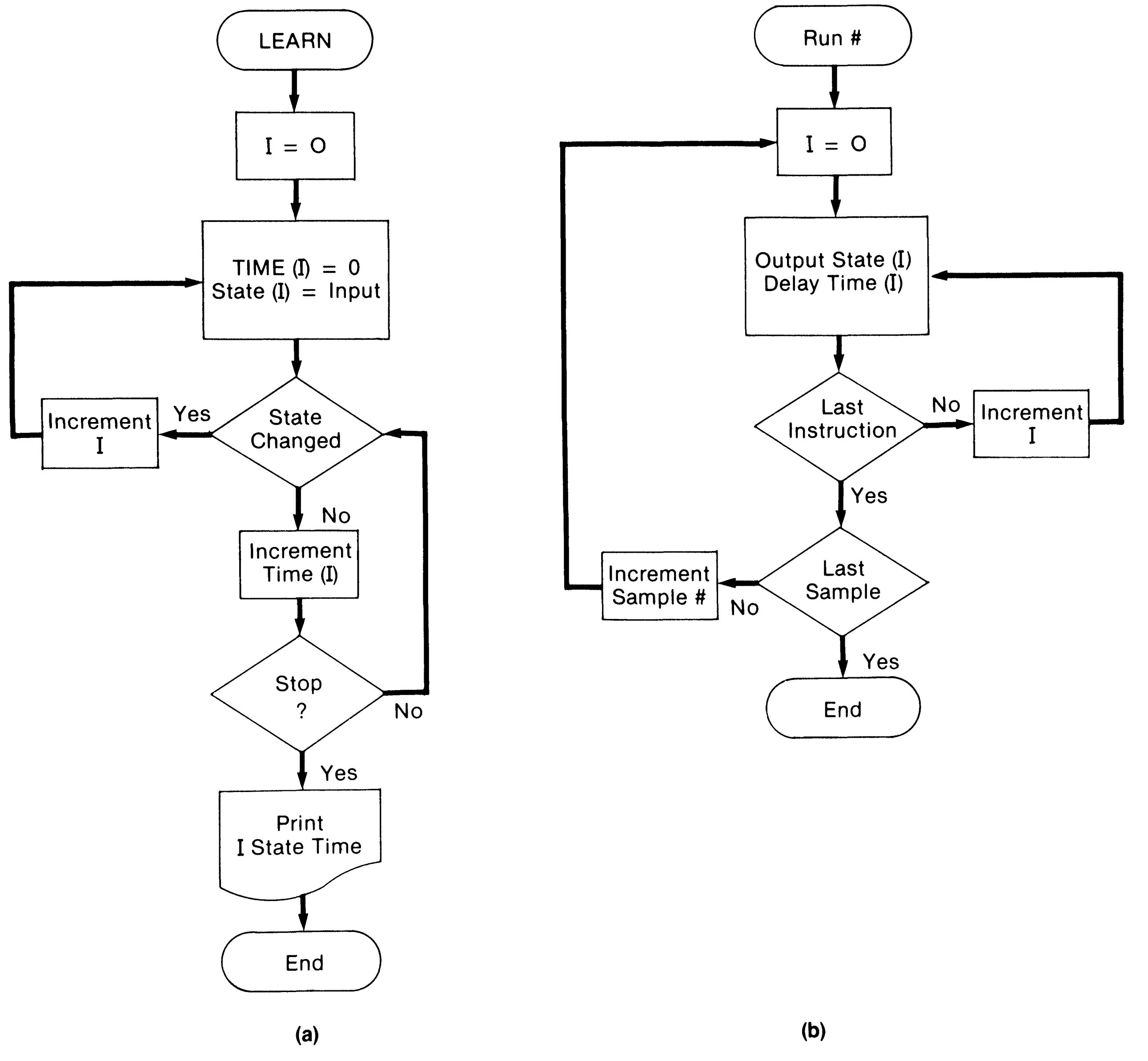

*Figure 4. Flow diagrams of the LEARN and RUN mode software are shown in parts (a) and (b) respectively.*

cedure. The method file can then be saved on cassette tape through a simple prompted procedure. Typing "RUN" and the number of samples is all that is required to duplicate the protocol for up to 99 samples.

Printed copies of the software are available upon request from the authors.

### Discussion

Today micro or minicomputers are often used to extend the capabilities of instruments. However, the user is immediately faced with the problems of connecting the microcomputer to the instrument in a way that doesn't damage or unintentionally interfere with its operation and, secondly, of telling the microcomputer how and when to take control. Traditional methods for implementing this type of control involve writing a program to interrogate the user about the analytical parameters and the delays after which they should change or, alternatively, an editor program which allows direct entry of commands into a methods file. Once this data is in the computer, a second program must interpret and execute these commands. Unfortunately, writing the programs for prompting the user and for editing, interpreting and executing the method file is an effort that must be repeated for each kind of instrument, since the functions and physical relationships differ widely. Furthermore, the physical interface is frequently complex and involves timing constraints and an understanding of instrument machine codes. The approach presented in this communication provides the analytical chemist with an alternate approach which is reasonably simple to implement yet is potentially powerful as a method of automation.

This communication has centred around the co-ordinated control of an "intelligent" system using time and the logic levels on parallel wires as the definition of state. The concepts discussed, however, clearly extend to systems involving only servos and to those employing other more complex ideas of state. Many instruments, for example, now communicate with their peripherals using serial ASCII codes, and streams of these characters and the delays between them could also be learned and reproduced by microcomputers. Other valid expressions of state include frequency, analogue voltage or more abstract events. In principle, the points of control for a system, whether "intelligent" or consisting only of pumps and valves, etc, must coincide and be electrically compatible with the microcomputer I/O. If this configuration can be realised, then the dependent and independent parameters of state can be linked in memory and reproduced as a digitised function.

The automation of vitamin $B_6$ analysis illustrates a solution to the common laboratory problem of synchronously controlling a modular analytical system without human attention (where the individual modules are from different suppliers). The method can be applied to many different kinds of systems without additional programming and it minimises the effort required in teaching microcomputers how to carry out an analytical procedure. The analyst presumably knows when and how to inject samples, to start integrating and to alter instrument parameters; what many find difficult, however, is telling the microcomputer how to control these functions.

ACKNOWLEDGMENT

The authors appreciate the contributions of Bruce Golden whose ideas were valuable in developing this concept of instrument automation.

DISCLAIMER

Mention of trademark or proprietary products do not constitute a guarantee or warranty of the product by the US Department of Agriculture and does not imply approval to the exclusion of other products that may also be suitable. It is the policy of the USDA not to endorse those commercial products used in the research over those not included in the research.

REFERENCES

[1] J.T. Vanderslice and C.E. Maire (1980),*J Chromatogr,* **196**, 176.

[2] J.T. Vanderslice, K.K. Stewart and M.M. Yarmas (1979), *J Chromatogr,* **176**, 200-205.

[3] J.T. Vanderslice, C.E. Maire, R.F. Doherty, and G.R. Beecher (1980), *J Agric Food Chem,* **28**, 1145.

[4] J.T. Vanderslice, C.E. Maire and G.R. Beecher (1981). In "Vitamin $B_6$ Analytical Methodology and Criteria for Assessing Nutritional Status. A Workshop, Timberline Lodge, Mt. Hood, Oregon, June 29-July 2, 1980. R. Reynolds and J. Lehlem, eds, Plenum Publ Corp, NY.

[5] J.T. Vanderslice, C.E. Maire and G.R. Beecher (May 1981), *Am J Clin Chem,* in press.

[6] J.T. Vanderslice, J.F. Brown, G.R. Beecher, C.E. Maire and S.G. Brownlee, *J. Chromatogr,* in press.

